# Bilateral axillary pigmented lesions in a melanoma patient

**DOI:** 10.1016/j.jdcr.2023.09.022

**Published:** 2023-10-02

**Authors:** Shannon Gurley, Jason Waldinger

**Affiliations:** a Chicago Medical School at RFUMS, North Chicago, Illinois; b Division of Dermatology, Department of Medicine, NorthShore University Health System, Evanston, Illinois

**Keywords:** BAP1 gene, melanoma, metastatic melanoma, ocular melanoma, pigmented lesions, uveal melanoma

## Case

**Fig 1:**
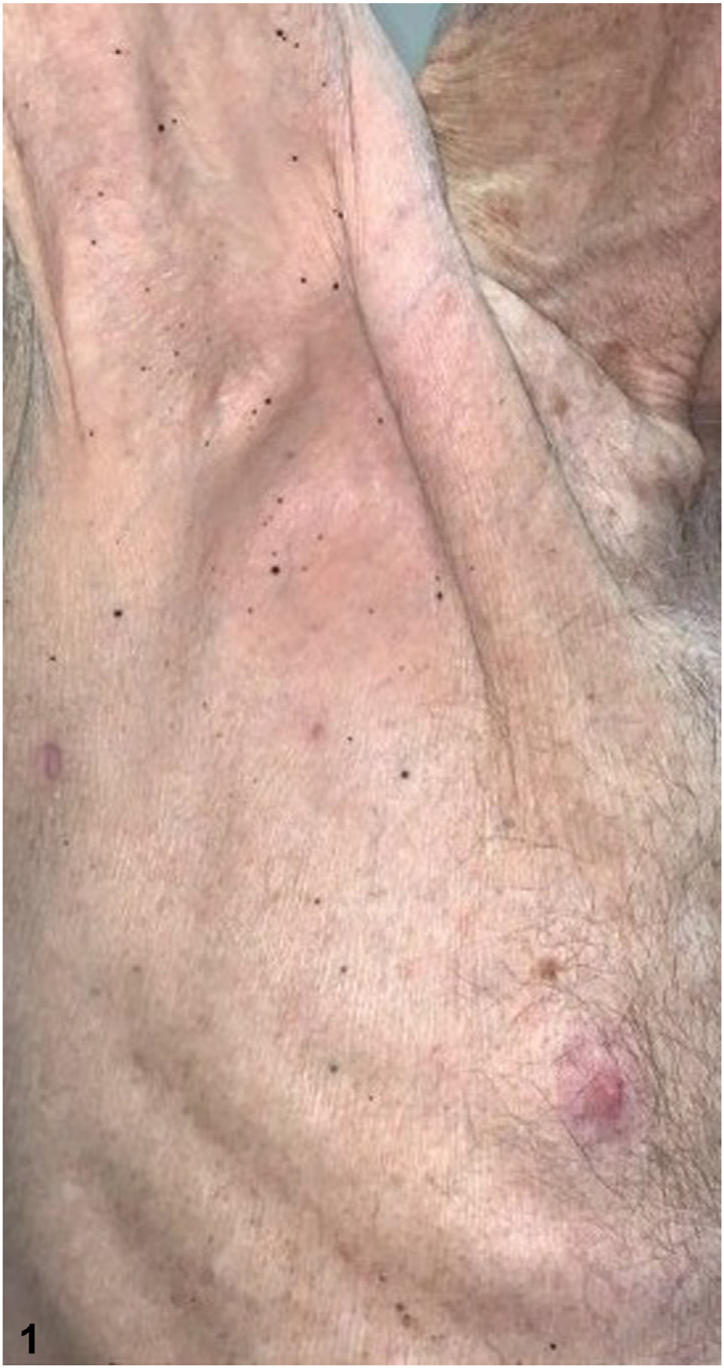

**Fig 2:**
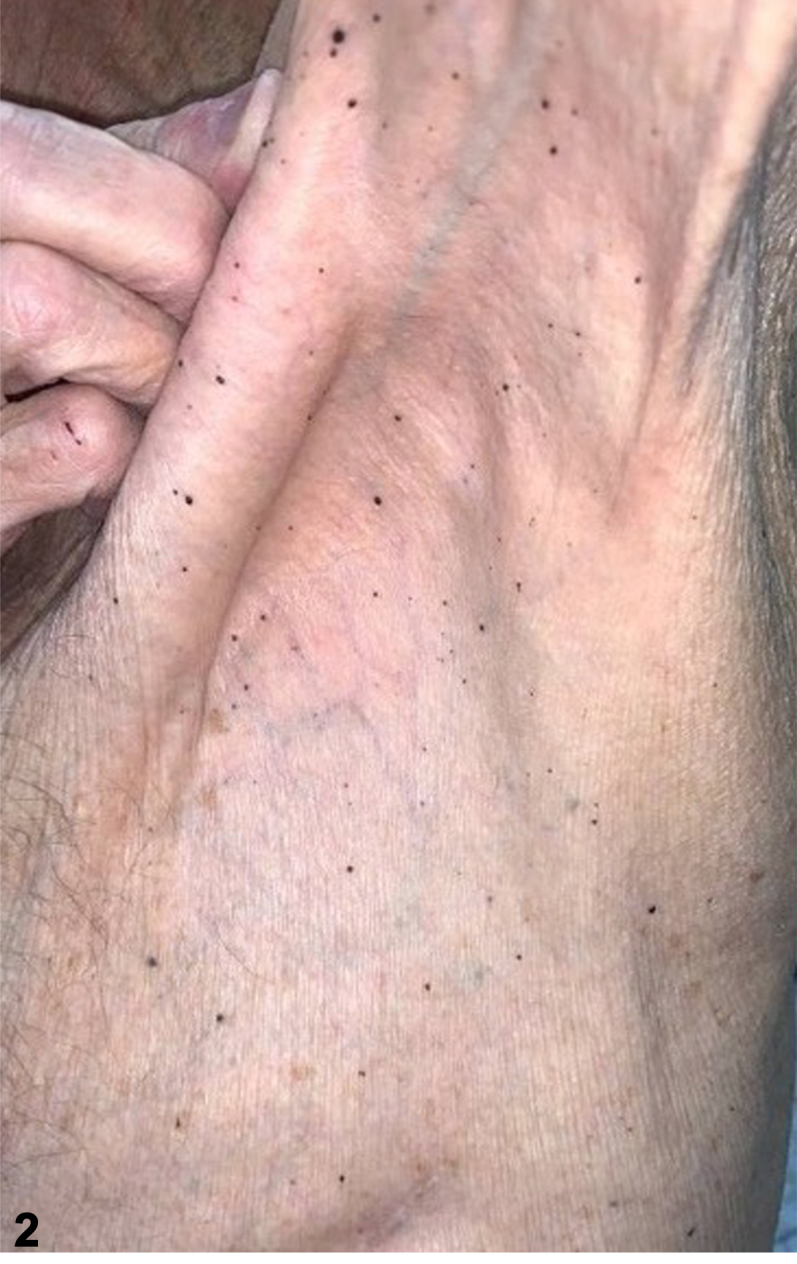

**Fig 3:**
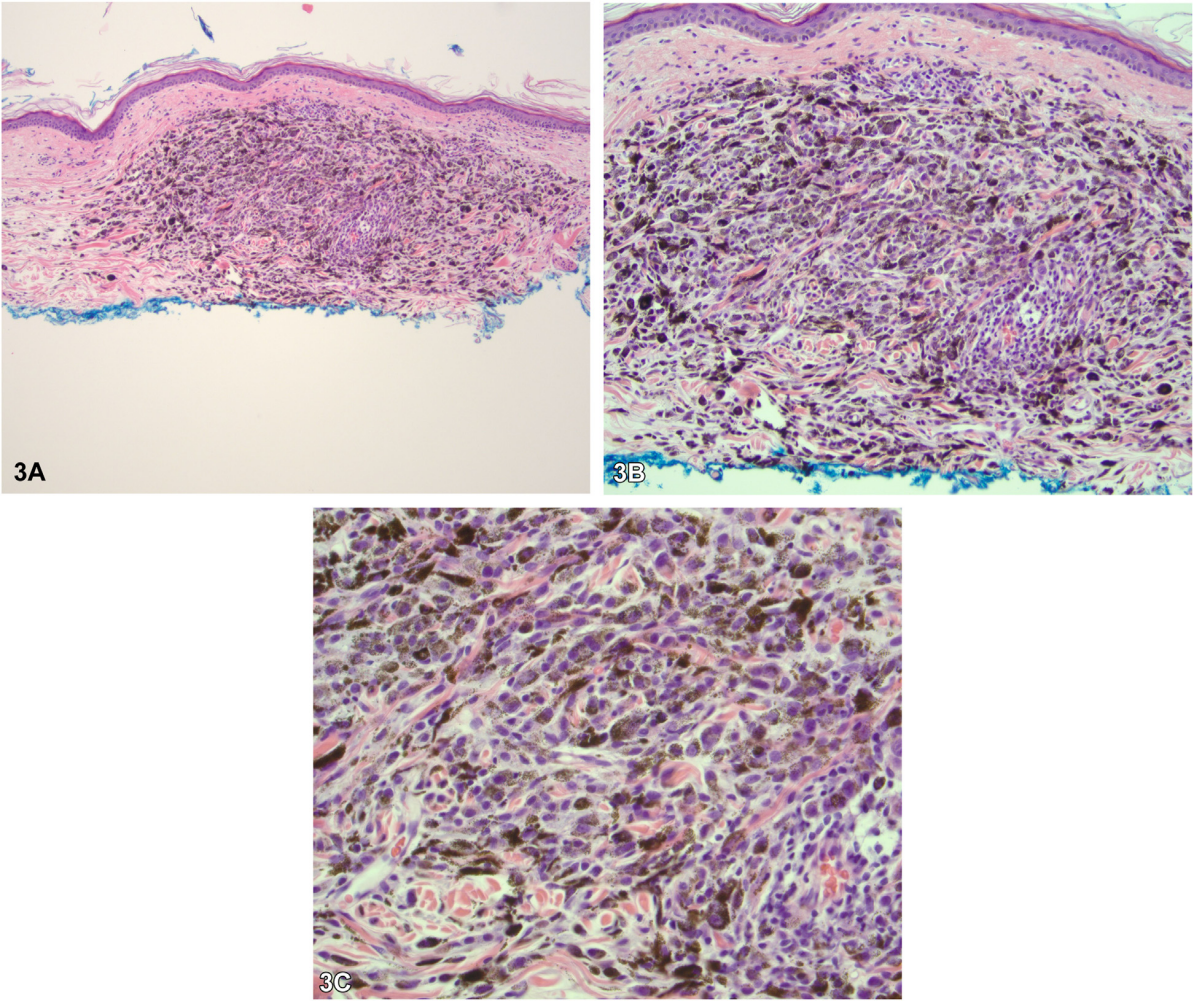

Our 84-year-old male patient has a history of cutaneous melanoma on the left abdomen in 1993 status-post wide local excision, and a 10 mm ocular melanoma of the right eye, in 2019 status-post plaque radiotherapy. In 2022 while receiving peptide receptor radionuclide therapy for pancreatic neuroendocrine cancer, he developed dozens of new 2-4 mm black macules on the bilateral shoulders, flanks, and axillae. Punch biopsies were positive for Mart1/Sox10 and PRAME expression and absent for p16 expression. Next generation sequencing showed a nonsense mutation within the BRCA1 associated protein (BAP1) gene and variants in the FGFR4, RET, and RB1 genes (Fig 1, Fig 2, Fig 3).

**Question 1: What is the most likely diagnosis?**

A. Pigmented basal cell carcinoma
B. Primary melanoma
C. Metastatic melanoma, cutaneous primary
D. Metastatic melanoma, uveal primary
E. Lentigines

**Answers:**

A. Pigmented basal cell carcinoma – Incorrect. Basal cell carcinoma typically presents as an isolated lesion and would not stain for melanocytic markers like Mart1/Sox 10.
B. Primary melanoma – Incorrect. Numerous new lesions distributed diffusely on the trunk are indicative of metastatic disease. The appearance of these macules are also distinct from the patient’s other nevi or lentigines.
C. Metastatic melanoma from cutaneous primary melanoma – Incorrect. Although clinical-pathologic correlation is consistent with a diagnosis of metastatic melanoma,^1^ the latency period of over 29 years reduces the likelihood that this presentation was caused by the patient’s cutaneous melanoma. Additionally, the BAP1 gene is associated with uveal melanoma more than cutaneous melanoma and uveal melanoma has a higher risk of metastasis compared to cutaneous melanoma.^2^
D. Metastatic melanoma from primary uveal melanoma – Correct. The shorter latency period of 3 years increases the likelihood that the primary ocular melanoma is responsible for widespread cutaneous metastases. Uveal melanoma is reported to metastasize in up to 50% of cases with the skin representing a common site for distant metastases.^2^ The BAP1 gene encodes a protein that removes a ubiquitin molecule to prevent protein degradation via the proteasome pathway thereby, avoiding apoptosis.^3^ The BAP1 gene mutations frequently occur in uveal melanoma and this nonsense mutation enables metastasis by upregulating cell-cell adhesion factors necessary for the cancer cells to breach the basement membrane.^3^
E. Lentigines – Incorrect. Lentigines are benign lesions that would not be consistent with the reported pathological findings.

**Question 2: The BAP1 gene is significant for its pathogenesis in BAP1-tumor predisposition syndrome. What other malignancy is associated with this syndrome?**

A. Pancreatic adenocarcinoma
B. Pancreatic neuroendocrine tumor
C. Malignant mesothelioma
D. Squamous cell carcinoma
E. Glioblastoma

**Answers:**

A. Pancreatic adenocarcinoma – Incorrect. Pancreatic adenocarcinoma is associated with familial melanoma and CDKN2A mutations but not BAP1 mutations.
B. Pancreatic neuroendocrine tumors – Incorrect. While our patient did have a primary neuroendocrine tumor of the pancreas, this is not related to BAP1-tumor predisposition syndrome.
C. Malignant mesothelioma – Correct. BAP1-tumor predisposition syndrome is an autosomal dominant disease that increases a patient’s risk of malignancies including uveal melanoma followed by the following neoplasms in decreasing order: mesothelioma, cutaneous melanoma, renal cell carcinoma, and basal cell carcinoma.^4^
D. Squamous cell carcinoma – Incorrect. Squamous cell carcinoma is not a malignancy associated within the BAP1-tumor predisposition syndrome.
E. Glioblastoma – Incorrect. Primary brain tumors are not associated with BAP1-tumor predisposition syndrome.

**Question 3: Which other type of melanoma is associated with BAP1 gene abnormalities?**

A. Mucosal melanoma
B. Melanoma ex blue nevus
C. Acral melanoma
D. Desmoplastic melanoma
E. Amelanotic melanoma

**Answers:**

A. Mucosal melanoma – Incorrect. This noncutaneous melanoma does not have an association with BAP1 gene mutations or deletions.
B. Melanoma ex blue nevus – Correct. Melanoma ex blue nevus or a malignant melanoma which arises within a pre-existing blue nevus has been shown to lack expression of the BAP1 gene and likely plays a role in the pathogenesis similarly to the mechanisms seen in uveal melanoma.^5^
C. Acral melanoma – Incorrect. While cutaneous melanoma is a part of the BAP1-tumor predisposition syndrome, this specific subtype has not been shown to have an association with BAP1 gene variations independent of the syndrome.
D. Desmoplastic melanoma – Incorrect. Desmoplastic melanoma is not associated with BAP1 gene abnormalities and does not necessarily have a specific known gene abnormality.
E. Amelanotic melanoma – Incorrect. There is not an association between BAP1 gene mutations or deletions in amelanotic melanoma.

## Conflicts of interest

None disclosed.
